# HIV Infection, Neurotoxicity, Inflammation, Premature Aging, and Therapeutic Challenges to PLWH: An Overview

**DOI:** 10.3390/ijms27052192

**Published:** 2026-02-26

**Authors:** Mudit Tyagi, Ulhas Naik, Kratika Tyagi, Madhulika Sharma, Gagan Kaushal, Alok Bhushan, Michael Bukrinsky, Priya Tyagi

**Affiliations:** 1 Department of Medicine, Center for Translational Medicine, Sidney Kimmel Medical College, Thomas Jefferson University, 1020 Locust Street, Jefferson Alumni Hall, Philadelphia, PA 19107, USA; ulhas.naik@jefferson.edu (U.N.); kratikatyagi28@gmail.com (K.T.); 2 Department of Internal Medicine, University of Kansas Medical Center, Kansas City, KS 66160, USA; msharma3@kumc.edu; 3 Department of Pharmaceutical Sciences, Jefferson College of Pharmacy, Thomas Jefferson University, Philadelphia, PA 19107, USA; gagan.kaushal@jefferson.edu (G.K.); alok.bhushan@jefferson.edu (A.B.); 4 Department of Microbiology, Immunology & Tropical Medicine, The George Washington University, Washington, DC 20037, USA; mbukrins@gwu.edu; 5 College of Computer, Mathematical, and Natural Sciences, University of Maryland, College Park, MD 20742, USA; ptyagi28@umd.edu

**Keywords:** HIV, neuroinflammation, neurotoxicity, neurocognitive disorders, aging, antiretroviral therapy, HAND

## Abstract

HIV infection remains a major global health challenge due to its complex pathogenesis and lifelong persistence in people living with HIV (PLWH). A central barrier to eradication is the virus’s ability to establish long-lived latent reservoirs in different tissues, including the central nervous system (CNS), where it evades immune clearance and antiretroviral therapy (ART). These reservoirs, seeded early during infection, fuel viral rebound if ART is interrupted, requiring lifelong treatment. In the CNS, HIV persists despite systemic viral suppression because of limited ART penetration across the blood–brain barrier (BBB), and infection of long-lived cells such as microglia and perivascular macrophages. Although modern ART regimens significantly reduce viral burden and HIV-related morbidity, they do not eliminate neurocognitive complications. Suboptimal CNS drug penetration and certain ART-associated toxicities contribute to CNS dysfunction, persistent neuroinflammation, and accelerated aging of the brain. As PLWH now experience increased life expectancy, prolonged exposure to ART and persistent low-level viral activity exacerbate chronic inflammation, immune activation, and metabolic dysregulation, collectively accelerating neurobiological aging. These pathological processes contribute to the development of HIV-associated neurocognitive disorders (HAND), which affect nearly half of virally suppressed PLWH. This review examines HIV-associated inflammation, neurotoxic pathways, and accelerated aging in PLWH in the modern ART era.

## 1. Introduction

HIV infection remains a significant global health challenge, not only due to its ongoing transmission but also because of the complex pathogenesis and the virus’s ability to persist lifelong in people living with HIV (PLWH). Since its discovery, extensive research has focused on developing virus-specific anti-HIV treatments that target the virus’s entry mechanisms and key enzymes, such as reverse transcriptase, integrase, and protease. These efforts culminated in the creation of therapy regimens, known as antiretroviral therapy (ART) or combination ART (cART). ART has become the cornerstone for treating HIV infection and has successfully reduced plasma HIV RNA levels to undetectable levels and significantly lowered AIDS-related mortality [1,2]. However, despite improvements in drug specificity and efficiency, ART is unable to eradicate HIV. Under a low metabolic state of the cell, HIV enters into a silent or latent state, where it is protected from both the host immune system and ART. However, latent HIV can be reactivated upon therapy interruption, leading to viral rebound, even in patients with undetectable plasma viremia [2,3,4]. Consequently, individuals infected with HIV are required to remain on lifelong medication to suppress the viral load. While ART can restore immune function to some extent, it is often incomplete and may lead to immune reconstitution inflammatory syndrome (IRIS) [5]. Over time, the episodes of suboptimal presence of ART and adaptation of HIV to the host’s immune system lead to the emergence of viral variants, reducing the efficacy of treatment. This often necessitates the replacement of drugs within the ART regimen [6,7,8,9]. Additionally, some ART medications can cause toxic side effects, making treatment intolerable for certain patients [10,11,12]. Furthermore, these drugs often have poor penetration into certain compartments, such as the central nervous system (CNS), limiting their effectiveness in these regions.

The CNS is considered an “immune-privileged” site, and the brain is often referred to as a sanctuary for HIV due to the restrictive regulation of cell migration and the selective permeability of the blood–brain barrier (BBB) and cerebrospinal fluid (CSF), which prevent antiretrovirals from effectively reaching the brain [11,13,14,15,16,17]. Various aspects of viral entry, transcription, and latency in the CNS are regulated by distinct mechanisms, enabling HIV-1 to persist in this compartment.

Although ART has dramatically reduced viral loads and HIV-related morbidity and mortality, it does not eliminate the risk of neurocognitive impairment [7,10,12,14,18,19]. Continuous use of ART is necessary for viral suppression, yet some ART drugs contribute to neurotoxicity, mitochondrial dysfunction, and may exacerbate age-related neuropathies. The suboptimal penetration of many ART drugs into the CNS allows sustained low-level viral replication and neuroinflammation, complicating HIV management [2,11,12,15,18,20]. As a result, PLWH live longer, but the prolonged use of ART and the persistence of HIV lead to significant systemic tolls, particularly in the CNS [11,14,15]. This causes accelerated neurotoxicity, neurocognitive impairments, and acquiring of premature age-associated comorbidities faster, such as heightened vulnerability to neuroinflammation, immune activation, metabolic disruptions, and epigenetic dysregulation [7,13,14,18,19,21,22,23]. Thus, neurotoxicities, including HAND, remain common, with nearly half of PLWH affected, despite viral suppression, ranging from mild cognitive impairment to severe dementia.

The neurotoxic effects of HIV are increasingly recognized as both direct and indirect, contributing to neurodegeneration [7,18,24,25,26,27]. The virus and its proteins, such as Tat and gp120, damage neural cells, disrupt BBB integrity, and induce chronic neuroinflammation, contributing to a spectrum of neurotoxicity [7,8,28,29,30]. This burden is especially pronounced among aging PLWH, who specifically experience accelerated brain aging, marked by early onset and greater severity of neurodegenerative changes like synaptic loss, white matter abnormalities, and the accumulation of pathological proteins like amyloid-β and tau [13,14,19,22,31,32,33,34,35,36].

The ongoing research is focused on strategies to enhance ART penetration into sanctuary sites and develop adjunctive therapies to mitigate inflammation and neurotoxicity. Biomarker development, advanced neuroimaging, and single-cell technologies are providing new insights pertaining to the underlying molecular mechanisms responsible for the pathogenesis of HAND [37]. This review highlights the current knowledge on the HIV life cycle, mechanisms of HIV-induced neurotoxicity, the interplay between HIV and aging, and the impact of ART. We discuss the benefits and limitations of current ART regimens, challenges, emerging therapeutic strategies, and future directions in the quest to improve the lives of PLWH by curbing age-associated comorbidities.

## 2. Global Burden and Epidemiological Overview

Since its identification in the early 1980s, HIV infection has evolved from a rapidly fatal disease to a chronic, manageable condition, largely due to the development and widespread implementation of ART. Despite remarkable advances in treatment and prevention, HIV remains a major global health burden [2,10,38]. According to the latest UNAIDS estimates, approximately 39 million people are living with HIV (PLWH) worldwide, with over 1.3 million new infections and nearly 700,000 AIDS-related deaths reported per year, [39,40,41]. Sub-Saharan Africa continues to bear a disproportionate share of the epidemic, but significant populations of PLWH reside in Asia, Eastern Europe, and the Americas, reflecting the global nature of the challenge [41,42]

The epidemiology of HIV is dynamic, shaped by evolving patterns of transmission, demographic shifts, and the impact of public health interventions. While the incidence of new infections has declined in many regions, key populations, including men who have sex with men, people who inject drugs, sex workers, and transgender individuals, remain at heightened risk [43,44]. The increasing age of the HIV-positive population, a testament to the success of ART, introduces new complexities related to comorbidities, polypharmacy, and the intersection of HIV with the biology of aging.

### 2.1. HIV Infection and Global Prevalence

HIV is a member of the Lentivirus genus within the Retroviridae family, characterized by a single-stranded, positive-sense RNA genome and a unique replication cycle involving reverse transcription and integration into the host genome. HIV infects cells that express both CD4 receptor and chemokine co-receptors (CCR5 or CXCR4). Given that chemokine receptors are ubiquitously expressed on different cell types, the expression of CD4 is the limiting factor to HIV infection. The main cell populations that express both receptor and coreceptor for HIV include T lymphocytes and monocytes/macrophages, which are the main targets of HIV in the system [45]. Upon infection, HIV rapidly establishes systemic viremia and replication-competent HIV proviral reservoirs throughout the body, with main sanctuary sites such as lymphoid tissues, lymph nodes, the gastrointestinal tract, and the CNS [8,18,46].

Globally, the overall prevalence of HIV among adults (aged 15–49) is about 0.7%, but this varies significantly by region. In sub-Saharan Africa, the prevalence is much higher, particularly among women, with HIV affecting 1 in 5 women in some countries. In contrast, regions like Europe and North America have seen declines in new infections due to widespread availability of ART, pre-exposure prophylaxis (PrEP), and harm reduction strategies. Sub-Saharan Africa bears the highest burden of the epidemic, accounting for approximately 65% of new infections globally, with young women aged 15–24 being particularly vulnerable due to factors like gender inequality, limited healthcare access, and higher rates of sexual violence [47,48,49].

### 2.2. Key Affected Populations

Several populations are disproportionately affected by HIV due to behavioral, social, and structural factors. Men who have sex with men (MSM), people who inject drugs (PWID), sex workers, and adolescent girls and young women are at higher risk due to unprotected sex, needle sharing, and unequal power dynamics in relationships. In sub-Saharan Africa, adolescent girls and young women face an especially high risk due to gender-based violence and lack of access to education and healthcare. Transgender individuals also experience high rates of HIV infection due to higher risk, stigma, and discrimination, which not only make them vulnerable but also hinder their access to healthcare services. These populations, along with others, contribute significantly to the global burden of HIV [47,48,50,51].

### 2.3. HIV Prevention, ART Access, and Challenges

Despite universal availability of ART, a significant number of PLWH globally are not receiving ART, leaving a significant gap, especially in low- and middle-income countries, where access, late diagnosis, stigma, and drug resistance remain major barriers. ART has proven effective in suppressing viral replication and reducing transmission, but challenges include late diagnosis, limited access to care, discrimination, and the emergence of drug resistance. Prevention strategies like PrEP, condom use, voluntary medical male circumcision, and mother-to-child transmission prevention have shown success, particularly in high-prevalence areas. However, stigma and discrimination continue to impede efforts, and there is an ongoing need for a functional cure, which remains a key focus of current research. Addressing these challenges is crucial for controlling the epidemic and improving health outcomes for PLWH [49,52,53].

Overall, the global burden of HIV remains substantial, with ongoing challenges in prevention, treatment access, and addressing the needs of key affected populations. While ART has made significant strides in controlling the epidemic, continued efforts are needed to reduce new infections, provide universal access to care, and address the social determinants of health that perpetuate the epidemic. The future of HIV control lies in improved prevention methods, better access to care, lasting impact of therapeutics, and breakthroughs in cure research, besides ensuring a holistic and equitable approach to the global HIV response.

### 2.4. Long-Acting Antiretrovirals (LA-ART)

Encouragingly, long-acting injectable antiretroviral therapies (LA-ART) have emerged as a major advancement in both HIV treatment and prevention, offering the potential to overcome long-standing challenges associated with daily oral regimens. The first complete injectable regimen, cabotegravir/rilpivirine (CAB/RPV; Cabenuva), achieves sustained viral suppression through monthly or bimonthly intramuscular administration. This approach provides an effective alternative for individuals with poor adherence to daily pills [54]. Growing evidence from clinical trials and real-world implementation highlights that LA-ART reduces pill fatigue, medication-related stigma, and daily treatment burden, thereby improving ART-acceptability among adolescents, socially marginalized populations, and individuals with unstable housing or mental health barriers [55,56]. The therapeutic landscape has further expanded with lenacapavir, the first-in-class HIV capsid inhibitor (Sunlenca/Yeztugo), administered as a twice-yearly subcutaneous injection. Results from Phase 3 PURPOSE prevention trials reveal >99.9% protection against HIV acquisition, while treatment studies show potent activity against multidrug-resistant HIV isolates and future potential for combination regimens requiring only semi-annual dosing [57,58,59]. These long-acting agents collectively hold promises for improving health equity by reducing dosing frequency, enhancing adherence, and alleviating stigma, the key barriers to successful HIV care [60,61]

### 2.5. Mechanistic Basis of Long-Acting Antiretroviral Therapies

The durability of LA-ART arises from the slow dissolution of drugs from depot formulations, extensive tissue and intracellular sequestration, macrophage-mediated redistribution, metabolic stability, and sustained pharmacodynamic activity, rather than from site-specific injection alone. The LA-ART is not simply a function of intramuscular or subcutaneous administration, but rather reflects a convergence of drug physicochemical properties, formulation science, tissue pharmacokinetics, and intracellular retention mechanisms. These features collectively enable sustained therapeutic drug levels over weeks to months following a single dose.

A defining characteristic of most LA-ART agents is their high lipophilicity and low aqueous solubility, which allows formulation as nanocrystalline suspensions. Drugs such as cabotegravir and rilpivirine are milled into nanoscale crystals that dissolve slowly at the injection site, creating a depot effect that continuously releases drug into the systemic circulation over extended periods [62,63,64]. Primarily, due to their lipophilic nature, LA-ART agents exhibit extensive tissue distribution, particularly into adipose tissue, lymphoid organs, and macrophage-rich compartments, resulting in slow redistribution back into plasma and prolonged terminal half-lives that can exceed 40–90 days [62,65]. Importantly, macrophages actively uptake nanocrystalline drug particles via phagocytosis, effectively serving as secondary depots that further extend drug persistence and facilitate trafficking to viral reservoir sites [66]. Thus, the slow dissolution kinetics of these nanocrystals, rather than the injection route per se, are a primary determinant of prolonged plasma exposure.

Additionally, metabolic stability plays a crucial role. LA-ART agents are deliberately engineered to undergo slow hepatic metabolism, often via glucuronidation rather than rapid oxidative pathways. Certain LA-ART drugs also exhibit intracellular trapping and slow efflux. These drugs accumulate within cells at concentrations exceeding plasma levels, where limited metabolism and slow cellular clearance sustain antiviral activity even as systemic concentrations decline [62,63,67,68]. Cabotegravir, for instance, is primarily metabolized by UGT1A1, a pathway associated with relatively slow clearance and minimal drug–drug interactions [62,63]. Similarly, lenacapavir possesses an exceptionally long intrinsic half-life due to its metabolic stability and slow systemic clearance, enabling dosing intervals of up to six months [69,70]. Moreover, lenacapavir further exemplifies how the molecular mechanism of action can contribute indirectly to long-acting efficacy. As a capsid inhibitor, lenacapavir binds with high affinity to conserved capsid interfaces and disrupts multiple stages of the HIV life cycle, including uncoating, nuclear import, and capsid assembly. This multi-stage inhibition allows sustained antiviral pressure even at low plasma concentrations, effectively extending its pharmacodynamic window [69,71].

Collectively, the prolonged efficacy of LA-ART reflects not merely its intramuscular delivery but a combination of favorable pharmacologic features, including slow release from depot formulations, extensive tissue and intracellular sequestration, macrophage-mediated redistribution, high metabolic stability, and sustained pharmacodynamic activity. Together, these properties enable durable drug exposure well beyond dosing intervals and fundamentally distinguish LA-ART from conventional oral antiretroviral regimens. A mechanistic understanding of these processes is essential for further optimizing next-generation long-acting agents, mitigating the risk of resistance, and extending long-acting strategies to anatomically protected compartments such as the CNS, as well as to cure-oriented interventions.

Notably, the same properties that confer prolonged durability also underlie the characteristic pharmacokinetic “tail” observed with LA-ART, in which sub-optimal therapeutic drug concentrations persist for weeks to months following discontinuation. Although this tail raises concerns regarding selection for drug resistance, it simultaneously highlights the exceptionally slow elimination kinetics that define these agents and enable infrequent dosing schedules. Careful management of this pharmacokinetic feature, through optimized initiation and discontinuation strategies, combination coverage, and therapeutic drug monitoring, will be critical to maximizing the clinical and preventive potential of long-acting antiretrovirals [55,72].

Despite these benefits, significant challenges remain. High cost, patent restrictions, and limited confidence in novel technologies currently hinder large-scale access, particularly in low-income regions where HIV burden is greatest [58]. Early implementation also requires intensive clinical follow-up and trained providers, further restricting use in resource-limited settings. Nonetheless, looking ahead, patent expiration, large-scale generic production, and infrequent dosing may substantially reduce cost, positioning LA-ART as a feasible global strategy for equitable HIV treatment and prevention. By shifting HIV care away from strict daily adherence and toward long-interval dosing, these innovations have the potential to transform long-term outcomes and quality of life for PLWH and those at HIV risk worldwide.

## 3. General Mechanisms of HIV-Mediated Neurotoxicity

With the widespread introduction of ART, the severe form of HIV-mediated neurotoxicity, historically manifested as HIV-associated dementia (HAD), has declined dramatically. This reduction reflects ART’s ability to suppress viral replication and limit the profound neuroinflammatory injury characteristic of untreated infection. However, despite these advances, neurocognitive impairment has not been eliminated. Instead, the clinical presentation has shifted toward a milder but highly prevalent spectrum of HIV-associated neurocognitive disorders (HAND), which persist even in individuals with long-term viral suppression [35,73,74,75,76,77,78]. Due to the lack of a transcriptional inhibitor, ART is unable to restrict HIV gene expression [2,45]. Persistent expression of viral RNA and proteins, including Tat, gp120, and Nef, within the brain sustains microglial activation, disrupts synaptic signaling, and induces synaptodendritic injury [18,34,79].

Neurodegeneration is a core characteristic of HAND; however, because neurons lack the canonical receptors required for HIV entry, they are not directly infected by the virus [2,18]. Instead, neurons sustain substantial bystander injury resulting from chronic exposure to neurotoxic viral proteins, inflammatory mediators, and oxidative stress generated by infected and exposed glial cells. Viral proteins such as Tat and gp120 have been shown to directly interact with neuronal NMDA receptors and chemokine receptors (CXCR4, CCR5), promoting excessive calcium influx, mitochondrial depolarization, and activation of apoptotic pathways [80,81,82,83]. Persistent inflammation driven by microglia and astrocytes leads to elevated levels of TNF-α, IL-1β, and reactive oxygen species (ROS), all of which impair synaptic function and its structural integrity. One hallmark of neuronal injury in HIV is synaptodendritic pruning, driven in part by complement system activation and microglial phagocytosis. This loss of synaptic connectivity underlies cognitive deficits associated with HAND. Moreover, neuronal energetic failure arises due to mitochondrial dysfunction, disrupted axonal transport, and reduced availability of neurotrophic factors such as brain-derived neurotrophic factor (BDNF). These changes diminish neuronal resilience and plasticity, contributing to memory impairment, slowed information processing, and executive dysfunction, which are commonly observed even in ART-treated individuals.

### 3.1. Reactive Free Radical Species and Neuronal Injury

Notably, during HIV infection, neuronal injury is not only driven by a single oxidative insult, but by a complex, self-reinforcing network of reactive species, collectively referred to as the reactive species interactome. This interactome encompasses reactive oxygen species (ROS), reactive nitrogen species (RNS), reactive sulfur species (RSS), lipid-derived reactive aldehydes, and reactive carbonyl species, all of which converge to disrupt neuronal homeostasis, mitochondrial integrity, and synaptic function. In PLWH, chronic immune activation, persistent viral protein expression, and ART exposure synergistically amplify this redox imbalance, contributing substantially to HAND.

### 3.2. Reactive Oxygen Species (ROS)

ROS, including superoxide (O_2_^−^), hydrogen peroxide (H_2_O_2_), and hydroxyl radicals (•OH), are central mediators of HIV-associated neurotoxicity. A number of HIV proteins have been shown to directly induce ROS generation by disrupting mitochondrial electron transport chain (ETC) complexes I and III, resulting in electron leakage and oxidative stress [79,84]. Activated microglia and infiltrating macrophages further amplify ROS production via NADPH oxidase (NOX2) activation, leading to oxidative damage in neighboring neurons [85,86].

### 3.3. Reactive Nitrogen Species (RNS)

Reactive nitrogen species, particularly nitric oxide (NO) and peroxynitrite (ONOO^−^), also play a vital role in HIV-associated neuronal injury. HIV Tat and gp120 induce inducible nitric oxide synthase (iNOS) expression in microglia and astrocytes, leading to excessive NO production [87,88,89,90]. NO rapidly reacts with superoxide to form peroxynitrite, a highly reactive molecule that nitrates tyrosine residues, damages mitochondrial DNA, and inactivates antioxidant enzymes such as manganese superoxide dismutase (MnSOD) [91,92]. Elevated nitrotyrosine levels have been detected in the CNS of PLWH with HAND, underscoring the pathological relevance of RNS [93].

### 3.4. Reactive Sulfur Species (RSS)

Emerging evidence implicates reactive sulfur species, including hydrogen sulfide (H_2_S) and persulfides, in HIV-associated neurotoxicity. Under physiological conditions, RSS act as neuromodulators and antioxidants; however, chronic inflammation and mitochondrial dysfunction shift sulfur metabolism toward toxic sulfide accumulation. HIV-induced mitochondrial impairment disrupts cysteine and glutathione metabolism, weakening redox buffering capacity and sensitizing neurons to oxidative and nitrosative stress [94,95,96,97]. Dysregulated RSS signaling further modulates NMDA receptor activity and mitochondrial respiration, compounding excitotoxic and bioenergetic injury in HAND.

### 3.5. Lipid Peroxidation and Reactive Aldehydes

ROS and RNS initiate lipid peroxidation of neuronal membranes rich in polyunsaturated fatty acids, generating highly toxic aldehydes such as 4-hydroxynonenal (4-HNE) and malondialdehyde (MDA). These lipid-derived reactive species form covalent adducts with proteins, impairing ion channels, synaptic receptors, and mitochondrial enzymes [98]. HIV Tat has been shown to enhance lipid peroxidation in neurons and astrocytes, while ART, particularly older NRTIs, were shown to further accelerate this process by inducing mitochondrial dysfunction [99,100]. Elevated 4-HNE adducts are observed in frontal cortex tissue from PLWH with cognitive impairment [101].

### 3.6. Reactive Carbonyl Species and Proteostasis Failure

Reactive carbonyl species (RCS), generated from glucose and lipid oxidation, contribute to carbonyl stress, leading to protein misfolding and aggregation. HIV-associated oxidative stress promotes the formation of advanced glycation end products (AGEs), which activate the receptor for AGEs (RAGE) on microglia and endothelial cells, reinforcing inflammatory signaling and BBB dysfunction [102]. Carbonyl stress impairs the ubiquitin-proteasome system and autophagy, processes already disrupted by HIV proteins, thereby accelerating accumulation of damaged mitochondria and neurotoxic protein aggregates [103,104].

### 3.7. ART-Associated Reactive Species Generation

Several antiretroviral agents, primarily NRTIs and some protease inhibitors, exacerbate reactive species production by inhibiting mitochondrial DNA polymerase-γ, leading to mtDNA depletion and defective oxidative phosphorylation [99,100]. This ART-induced mitochondrial stress amplifies ROS generation and sensitizes neurons to injuries mediated by viral proteins. Although newer ART regimens are less toxic, cumulative exposure over decades may still contribute to redox imbalance, especially in aging PLWH.

### 3.8. Integrated Reactive Species Interactome in Neuronal Injury

Collectively, ROS, RNS, RSS, lipid-derived aldehydes, and carbonyl species form an interconnected redox network that drives neuronal dysfunction. These reactive species reinforce one another, impair antioxidant defenses, destabilize mitochondria, disrupt synaptic signaling, and activate apoptotic pathways. Importantly, this interactome operates downstream of both viral persistence and chronic inflammation, making it a critical mechanistic link between HIV infection, ART exposure, and accelerated neurodegeneration in PLWH. Thus, excess free radicals oxidize lipids, proteins, and nucleic acids, impairing synaptic plasticity and triggering the opening of the mitochondrial permeability transition pore (mPTP), which ultimately activates intrinsic apoptotic pathways. Moreover, in postmortem brains from PLWH, the high prevalence of elevated markers of free radical-mediated damage and their correlation with the severity of cognitive impairment are frequently noted [101,105].

### 3.9. HIV-Mediated “Direct” and “Indirect” Mechanisms of Neurotoxicity

**Figure 1:**
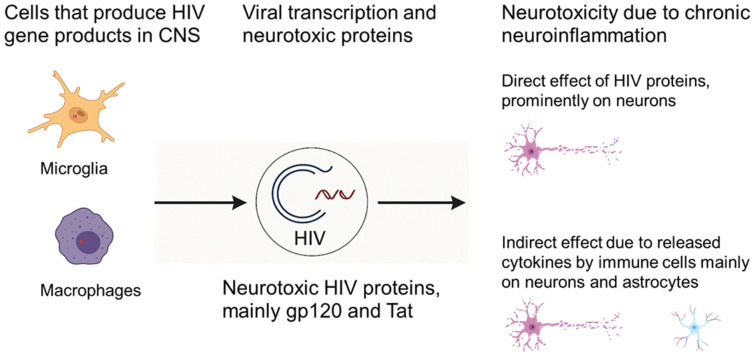
HIV induces neuronal dysfunction and bystander injury (NDBI) through a combination of direct and indirect mechanisms. Direct neurotoxicity arises from HIV gene products, while indirect damage results from proinflammatory cytokines, chemokines, and other neurotoxic mediators released predominantly by activated immune cells. Within the CNS, HIV establishes productive or persistent infection primarily in microglia, perivascular macrophages, and infiltrating monocyte-derived macrophages. These infected cells secrete a range of neurotoxic viral proteins, including Tat, gp120, and Nef, which diffuse through the brain parenchyma and act on neighboring neurons as well as astrocytes, oligodendrocytes, and additional glial populations. Concurrently, activated CNS immune cells release proinflammatory cytokines, reactive oxygen species, and other toxic metabolites that amplify local inflammation. Together, these viral and host-derived factors drive synaptodendritic injury, mitochondrial dysfunction, oxidative stress, glutamate excitotoxicity, and the activation of apoptotic signaling pathways, culminating in neuronal degeneration and loss of neural connectivity. The cumulative and sustained impact of viral protein exposure and neuroimmune activation forms the pathological basis for the development and progression of HAND. The figure was created with BioRender.com.

There are two general mechanisms through which HIV can inflict Neuronal Dysfunction and Bystander Injury (NDBI). One of the mechanisms is the “direct mechanisms”, where various viral proteins initiate neuronal injury and death. There is strong evidence in support of direct injury through HIV proteins, primarily gp120 and Tat. Numerous studies have shown the neurotoxic nature of these HIV proteins in the CNS [18,28,30,33,34,106,107,108,109]. These proteins are well characterized, and a large amount of literature has been published describing the mechanisms through which different viral proteins mediate neurotoxicity [110] (Figure 1). On the other hand, “indirect mechanisms” involve homeostatic disruption mainly of the host immune system, as immune cells are primarily impacted by HIV, such as host macrophages and microglia, which get activated and release various factors, either through interaction with viral proteins or by immune stimulation, that contribute to neurodegeneration. Common molecular pathways implicated in triggering neuronal apoptosis include Ca^2+^ overload; activation of p38 MAPK and p53; activation of cell cycle proteins and caspases; free radical formation; lipid release and peroxidation; and chromatin condensation [111,112,113,114,115,116,117].

HIV exerts neurotoxic effects on the CNS through a combination of direct actions by viral proteins and indirect mechanisms involving chronic neuroinflammation. The neurotoxicity driven by the direct actions of viral proteins on neuronal and glial cells disrupts cellular homeostasis, induces oxidative stress, and activates apoptotic pathways. The indirect mechanism that impacts neurotoxicity primarily involves HIV-induced neuroinflammation driven by sustained activation of CNS-resident and infiltrating immune cells, which create a toxic milieu that perpetuates neuronal injury. Together, these processes disrupt neuronal and glial cell homeostasis, drive oxidative stress, activate apoptotic pathways, and promote neuronal injury and loss, contributing to the spectrum of HAND [118,119]. Most neuronal damage in HAND is thought to occur via indirect mechanisms, as immune activation in the CNS sets off a cascade of inflammation and cellular stress that ultimately impairs neural circuits and cognitive function [80,120].

#### 3.9.1. Direct Mechanisms of Neurotoxicity

HIV neurotoxicity is driven by the direct actions of viral proteins on neuronal and glial cells, disrupting cellular homeostasis, inducing oxidative stress, and activating apoptotic pathways. These mechanisms contribute to synaptic loss, dendritic pruning, and neuronal death, underpinning HAND. Below, we detail the roles of key viral proteins and their impact on mitochondrial function.

Viral proteins-mediated neuronal injury:

As discussed above, the HIV proteins gp120 and Tat are principal mediators of HIV-associated neurotoxicity, as both disrupt essential cellular signaling pathways [18,46,121,122]. These viral proteins engage distinct yet convergent mechanisms, including calcium dysregulation, oxidative stress, inflammatory signaling, and transcriptional reprogramming, that ultimately compromise neuronal viability and synaptic function. For example, …

Neurotoxicity mediated by gp120:

This envelope glycoprotein binds to neuronal chemokine receptors (CCR5, CXCR4), triggering intracellular signaling cascades that lead to excessive calcium influx, oxidative stress, and apoptosis. gp120 also impairs astrocytic glutamate uptake, promoting excitotoxicity and further neuronal injury [109,112]. Key mechanisms include:

Glutamate Excitotoxicity: The gp120 reduces astrocytic glutamate reuptake by downregulating excitatory amino acid transporters (EAAT-1/2), leading to extracellular glutamate accumulation and overactivation of neuronal NMDA receptors (NMDARs) [73]. Additionally, excess NMDAR activation triggers calcium influx, mitochondrial calcium overload, and reactive oxygen species (ROS) production, culminating in oxidative stress and apoptosis [123].

Arachidonic Acid Release: HIV envelop (gp120) by stimulating arachidonic acid release inhibits neuronal and astrocytic glutamate reuptake, exacerbating excitotoxicity [124,125].

Mitochondrial Dysfunction: The gp120 has also been shown to alter mitochondrial dynamics, promoting fusion (elongated mitochondria) and impairing respiration. This disrupts ATP production and increases ROS, leading to neuronal energy deficits and oxidative damage [126,127].

BBB Disruption: By upregulating matrix metalloproteinases (MMPs), gp120 has been shown to promote the degradation of tight junction proteins (claudin-5, occludin) and increase BBB permeability, facilitating neuroinvasion of toxins and immune cells [123,128].

Neurotoxicity mediated by Tat:

The Tat protein is secreted by infected cells and taken up by neurons and astrocytes. Tat potentiates NMDA receptor activity, leading to calcium overload, mitochondrial dysfunction, and increased production of ROS. It also activates pro-inflammatory signaling pathways, such as p38 MAPK, and promotes the release of neurotoxic cytokines [81,82,83,109,126]. Key mechanisms include:

NMDAR Potentiation: Tat binds lipoprotein receptor-related protein (LRP) on neurons, activating Src kinase to phosphorylate NMDAR subunits (NR2A/NR2B). This enhances calcium influx, leading to mitochondrial dysfunction and ROS overproduction [90]. In addition, Tat regulates biphasic calcium dynamics. Initially, Tat potentiates NMDAR-evoked calcium flux but later induces adaptation via nitric oxide synthase (NOS)/soluble guanylate cyclase (sGC) pathways, which may fail under chronic exposure, leading to irreversible damage [81,82,83,129,130].

Oxidative Stress: Tat has been convincingly shown to promote neurotoxicity by increasing mitochondrial ROS by impairing electron transport chain (ETC) complexes I and IV, reducing ATP synthesis, and promoting cytochrome c release, a precursor to apoptosis [123,126].

Synaptic Injury: Tat has been shown to promote synaptic injury by activating p38 MAPK in neurons and microglia, triggering pro-apoptotic signaling (e.g., caspase-3 activation) and synaptic protein degradation [112].

Other HIV proteins, such as gp41, Vpr, and Nef, also contribute to CNS injury by disrupting cellular homeostasis, impairing mitochondrial function, and triggering neuronal apoptosis. The envelope transmembrane protein gp41 has been shown to induce excessive production of nitric oxide (NO) through activation of nitric oxide synthase, leading to oxidative stress and downstream neurotoxicity. The accessory protein Vpr perturbs mitochondrial integrity by disrupting the mitochondrial membrane potential, promoting the opening of the mitochondrial permeability transition pore, and facilitating cytochrome c release, thereby activating intrinsic apoptotic pathways. Meanwhile, Nef interferes with autophagy and mitochondrial quality-control mechanisms, resulting in the accumulation of damaged mitochondria, altered cellular metabolism, and heightened susceptibility to inflammatory and oxidative stress-mediated injury. Together, these viral proteins amplify neuroinflammatory cascades and exacerbate neuronal dysfunction, contributing to the persistence of HAND even under suppressive ART [33,109,131].

Collectively, the CNS serves as both a site of a stable HIV reservoir and a locus of chronic inflammation, contributing significantly to the neurological and psychiatric complications of HIV infection. Understanding the molecular mechanisms that sustain neuroinflammation is therefore critical to designing strategies to target CNS reservoir, neurotoxicity, and the premature aging process.

#### 3.9.2. Indirect Mechanisms of Neurotoxicity

##### HIV and CNS Inflammation

Acute and early stages of HIV infection trigger robust systemic inflammation characterized by widespread immune activation and cytokine release. Current ART regimens are highly effective in suppressing plasma viremia and substantially controlling systemic inflammation. However, despite sustained peripheral viral suppression, residual inflammation persists, especially within the CNS, a particularly vulnerable anatomical site. Even in well-controlled individuals living with HIV who maintain undetectable plasma viral loads under long-term ART, inflammatory activity in the CNS often lingers [7,18,132].

HIV persistence in the CNS is largely attributed to the restricted penetration of ART across the BBB, pharmacokinetic differences in CNS microenvironments, and the presence of immune-privileged compartments within the CNS, which allow low-level viral persistence and intermittent replication [133]. Furthermore, fluctuating or suboptimal ART concentrations in both the CNS and peripheral compartments contribute to ongoing immune activation and the development of several HIV-associated non-AIDS (HANA) comorbidities, including cardiovascular disease, cancer, renal impairment, metabolic dysfunction, neurocognitive decline, and signs of accelerated biological aging [7,10,18,19,46,134,135,136,137]. Chronic inflammation in the CNS creates a state of persistent low-level inflammation that is usually known as “inflammaging”, which predisposes PLWH to early-onset of different neuropathies, including neurodegeneration and vascular dysfunction leading to neurotoxicity and HAND. Additionally, certain HIV gene products, primarily Tat and gp120 proteins, disturb immune balance, foster abnormal cytokine production, and induce oxidative stress, which together lead to neuronal injury and compromise the integrity of the BBB. Continued stimulation of microglia and astrocytes within the CNS perpetuates a cycle of chronic inflammation and neurodegenerative changes, persisting even among well-controlled PLWH with undetectable HIV levels in plasma.

Activation of the innate and adaptive immune systems plays a central role in regulating inflammatory responses. Their dysregulation results in a heightened immune response, marked as inflammation. In the brain, inflammation primarily depends on the HIV infection of microglia, the CNS tissue-resident macrophages, and perivascular macrophages. Similarly, exposure to HIV and its gene products, which are present in the CNS milieu of PLWH, to astrocytes is recently recognized as another crucial immune component in the CNS that disturbs the CNS homeostatic immune-response balance. The imbalanced immune responses modulate the release of cytokines (both pro- and anti-inflammatory functions) by the CNS immune cells, which is vital for cellular homeostasis and cell health. The chronic inflammation, primarily due to persistent elevation of proinflammatory cytokines, results in tissue damage and contributes to neurodegeneration and different kinds of neuropathies [13,18,22,46,138,139]. Thus, understanding the molecular pathways linking HIV, inflammation, and the balance of pro- and anti-inflammatory cytokines is crucial for developing strategies to mitigate long-term neurological complications, a major contributor to premature aging, and improve the quality of life for PLWH.

As the principal regulatory center of the body, dysfunction within the CNS compromises overall physiological function. Accordingly, CNS inflammation exerts distinct and long-lasting effects on health outcomes. The CNS serves both as a sanctuary for persistent HIV and a hub for chronic immune activation. Despite effective systemic viral suppression, HAND remains prevalent in nearly 50% of PLWH. Ongoing activation of HIV-infected microglia, perivascular macrophages, and astrocytes sustains the production of proinflammatory cytokines, including IL-1β, TNF-α, and IFN-γ, leading to synaptic injury and disruption of neuronal networks [13,18,140].

Persistent neuroinflammation is recognized as a hallmark of HIV neuropathogenesis and the aging process, resulting from both direct viral effects and indirect immune responses. Importantly, the aging process further promotes these adverse effects, as aged PLWH face accelerated immunosenescence and neurodegenerative changes that mirror conditions like Alzheimer’s disease [141,142]. Thus, the intersection of HIV infection, systemic inflammation, and CNS-specific neuroinflammation is central to promoting and understanding HIV-associated comorbidity in the era of ART.

## 4. Chronic Neuroinflammation

Shortly after systemic infection, HIV can be detected in the cerebrospinal fluid (CSF), often within days to weeks, indicating early neuroinvasion [143]. Viral entry into the CNS primarily occurs through infected monocytes and CD4^+^ T cells that cross the BBB [2,144]. Once within the CNS, HIV establishes infection predominantly in microglia and perivascular macrophages, which serve as long-lived viral reservoirs capable of sustaining low-level replication even under suppressive ART [145,146].

HIV infection in the CNS initiates a cascade of immune activation and cytokine release, driving chronic neuroinflammation and neuronal injury. Although ART effectively suppresses viral replication in the periphery, its efficacy in the CNS is limited because of restricted drug penetration across the BBB and pharmacokinetic differences in CNS microenvironments [133]. Persistent expression of viral RNA and proteins within the brain sustains microglial activation, disrupts synaptic signaling, and induces synaptodendritic injury [18,34,79]. Thus, HIV-induced neuroinflammation arises from a complex interplay between direct infection or exposure of CNS-resident cells, infiltration of peripheral immune cells, and persistent release of viral proteins. This multifactorial disruption leads to a functional imbalance among neurons, astrocytes, microglia, and endothelial cells, resulting in cytokine dysregulation, oxidative stress, and mitochondrial dysfunction within the CNS. The resultant inflammatory cascade, characterized by microglial activation, astrocyte reactivity, BBB disruption, and neuronal injury, collectively drives chronic neuroinflammation. These processes trigger a range of neuropathies, including HAND and other neurodegenerative manifestations, even in the era of effective ART.

In short, HIV neuropathogenesis reflects a complex, multicellular interplay among microglia, astrocytes, neurons, endothelial cells, and infiltrating monocyte-derived macrophages. Each cell type contributes uniquely to the initiation, amplification, and maintenance of neuroinflammation within the CNS.

### 4.1. Microglia, the Primary Driver of Neuroinflammation

HIV-infected or chronically immune-activated microglia play a central role in sustaining neuroinflammation within the CNS. Upon exposure to HIV or its viral proteins, microglia secrete high levels of pro-inflammatory cytokines such as TNF-α, IL-1β, and IL-6, along with chemokines including CCL2 and CXCL10, which further recruit and activate additional immune cells and amplify local inflammatory signaling [147,148,149]. A major downstream driver of this heightened activation is the NLRP3 inflammasome, which is robustly stimulated by HIV proteins, particularly Tat. Activation of NLRP3 leads to caspase-1-mediated pyroptosis and the subsequent release of potent inflammatory mediators IL-1β and IL-18, thereby intensifying tissue injury and promoting a self-perpetuating inflammatory cycle within the CNS [92,147,149].

Beyond cytokine and inflammasome signaling, activated microglia also generate substantial amounts of reactive oxygen species (ROS) and nitric oxide (NO) through NADPH oxidase and inducible nitric oxide synthase pathways. This oxidative burden disrupts neuronal mitochondrial function, impairs ATP production, and enhances susceptibility to excitotoxicity and apoptosis. Together, these processes contribute to progressive synaptic dysfunction, dendritic injury, and neuronal loss, which collectively promote the development and persistence of HAND despite long-term ART-mediated viral suppression [24,150].

### 4.2. Perivascular Macrophages and Infiltrating Monocytes, Another Key Contributor to Neuroinflammation

Perivascular macrophages are another CNS cell population that is a HIV target and is an additional major HIV reservoir within the CNS. Recent findings indicate that, similarly to microglia, the perivascular macrophage population also has a prenatal origin from the yolk sac [151,152,153]. These cells express high levels of CCR5, facilitating infection and viral persistence. Once infected or activated, they secrete a broad range of pro-inflammatory cytokines (e.g., TNF-α, IL-1β, IL-8, and MCP-1) and chemokines (e.g., CCL2, CXCL10) that not only recruit more immune cells but also disrupt BBB tight junctions, promoting further viral entry into the CNS and perpetuating local inflammation [154,155]. Perivascular macrophages interact closely with endothelial cells and microglia, amplifying local inflammation and oxidative stress. Like microglial cells, the relatively long lifespan of perivascular macrophages allows them to maintain infection and secrete neurotoxic mediators for extended periods, perpetuating neuroinflammation even when systemic viremia is suppressed.

Perivascular macrophages also interact with endothelial cells at the BBB, releasing MMP-2 and MMP-9, which degrade basement membrane and tight junction components, thereby worsening barrier permeability. Their production of ROS and nitric oxide further contributes to oxidative stress and neurovascular injury. Additionally, because perivascular macrophages undergo slow turnover, they can harbor HIV for extended periods, even during effective ART. Their ability to self-renew locally enables ongoing release of viral RNA, proteins, and extracellular vesicles, all of which promote chronic inflammation and neuronal damage. Thus, HIV infection of perivascular macrophages contributes to both neurotoxicity and CNS reservoirs.

### 4.3. Astrocytes, the Amplifiers and Modulators of Neurotoxicity

Astrocytes mainly experience exposure to HIV and its gene products, as these cells are rarely infected with HIV, and mostly remain non-productive of infectious HIV; yet, they are major contributors to HIV-induced neuroinflammation through reactive astrogliosis.

Astrocytes, although not major producers of infectious HIV, undergo significant functional alterations in response to viral proteins (mainly gp120 and Tat) and inflammatory cytokines (e.g., IL-1β, TNF-α), which induce astrocytic activation, leading to increased expression of GFAP, release of IL-6, CCL2, and CXCL10, and loss of glutamate-buffering capacity [156,157].

One of the most detrimental consequences, but the relative importance is unknown, is the downregulation of excitatory amino acid transporters EAAT-1 and EAAT-2, which are essential for clearing extracellular glutamate from synaptic spaces [92,150]. Impaired glutamate uptake leads to extracellular glutamate accumulation, driving NMDA receptor overactivation and consequent excitotoxic neuronal injury, a hallmark mechanism contributing to HAND.

In addition to excitotoxicity, dysfunctional astrocytes adopt a proinflammatory phenotype, releasing cytokines and chemokines such as IL-6, CCL2, and other mediators that perpetuate microglial activation and neuroimmune signaling [92,137]. Astrocytes also lose their ability to regulate potassium (K^+^) homeostasis, a critical function for maintaining neuronal membrane potential and synaptic stability. Disruption of K^+^ buffering further destabilizes neuronal networks and increases vulnerability to oxidative and inflammatory stress.

Notably, reactive astrocytes secrete matrix metalloproteinases (MMP-2, MMP-9), which degrade endothelial tight-junction proteins and compromise BBB integrity [31,158,159,160]. Astrocytes also maintain bidirectional signaling with microglia: microglial cytokines enhance astrocyte reactivity, while astrocyte-derived chemokines further recruit and activate microglia to create a self-perpetuating inflammatory feedback loop. Moreover, astrocytes propagate inflammatory signals through gap junction channels, allowing the rapid spread of calcium waves, reactive oxygen species, and toxic metabolites across astrocytic networks. This gap junction-mediated dissemination significantly amplifies neuroinflammation and extends neuronal dysfunction beyond the initial site of injury, thereby facilitating widespread CNS involvement in HIV-associated neurotoxicity [92]

Together, these alterations transform astrocytes from supportive glial cells into active contributors to neuroinflammation, excitotoxicity, and synaptic dysfunction, highlighting their central role in the progression of HAND.

### 4.4. Endothelial Cells, the Gatekeepers of the Neurovascular Unit

The brain microvascular endothelial cells (BMECs) that form the structural basis of the BBB are critical regulators of CNS homeostasis. HIV proteins such as Tat, gp120, and Nef, along with inflammatory cytokines produced by activated glia, profoundly disrupt endothelial function. Exposure to these viral proteins leads to downregulation and mislocalization of tight-junction proteins, including claudin-5, occludin, and ZO-1, resulting in increased BBB permeability [159,160]. This structural compromise facilitates the entry of infected monocytes and inflammatory mediators into the CNS, amplifying neuroinflammation. Endothelial cells also undergo activation, characterized by increased expression of ICAM-1, VCAM-1, and E-selectin, which promote adhesion and transmigration of leukocytes [46,159,161]. Furthermore, HIV proteins induce oxidative stress in endothelial cells through activation of NADPH oxidase and mitochondrial dysfunction, leading to apoptosis and microvascular instability. These processes impair cerebral blood flow, damage the neurovascular unit (NVU), and contribute to cerebral small vessel disease (CSVD) commonly observed in PLWH. Moreover, compounding this injury, endothelial cells release pro-inflammatory cytokines and chemokines that further propagate microglial and astrocytic activation. Together, these events weaken the protective role of the BBB and promote a cycle of inflammation, infiltration, and neurovascular dysfunction that exacerbates neuronal injury in HIV infection.

### 4.5. Oligodendrocytes, the Protectors of Myelin Integrity

The exposure of HIV proteins and inflammatory cytokines also affects oligodendrocytes, reducing myelin gene expression and promoting demyelination. Tat and gp120 induce ER stress and apoptosis in oligodendrocyte precursor cells, leading to white matter abnormalities frequently observed in neuroimaging studies of PLWH [27,149,162]. Disrupted myelination contributes to slowed cognitive processing and impaired neuronal conduction/signaling.

### 4.6. Neurons, the Primary Targets of Bystander Damage

Neurons lack HIV receptors; thus, they are not infected by HIV but suffer extensive bystander injury due to viral proteins and inflammatory mediators. Tat and gp120 directly interact with neuronal NMDA receptors and chemokine receptors (CXCR4/CCR5), causing calcium overload, mitochondrial dysfunction, and apoptosis [80,83,163]. Prolonged exposure to TNF-α, IL-1β, and oxidative stress leads to synaptic pruning and dendritic spine loss, which correlate with cognitive impairment. Furthermore, decreased BDNF levels and impaired axonal transport compromise neuronal plasticity, reinforcing the neurodegenerative component of HAND [164,165].

In essence, neurons become collateral victims of a broad bystander toxicity cascade that begins with the release of HIV viral proteins and is perpetuated by persistent glial activation and neurovascular dysfunction. Although neurons are not productively infected by HIV, they are continuously exposed to a hostile microenvironment characterized by excitotoxic signaling, oxidative stress, mitochondrial impairment, and inflammatory cytokines released from activated microglia, astrocytes, and infiltrating macrophages. Simultaneously, disruption of the BBB and neurovascular unit facilitates the entry of additional inflammatory mediators and infected cells, further destabilizing neuronal homeostasis. Over time, this combination of indirect insults drives synaptic loss, dendritic retraction, impaired plasticity, and ultimately neuronal degeneration, which collectively underpin the cognitive and functional deficits observed in HAND.

Altogether, these cell-specific responses establish a chronic, self-reinforcing inflammatory network within the CNS. Microglia and macrophages serve as viral reservoirs and primary cytokine sources; astrocytes amplify and sustain inflammation; endothelial dysfunction facilitates peripheral immune cell entry; and neurons experience and primarily manifest progressive bystander damage. This integrated cellular pathology underlies the persistence of neuroinflammation and cognitive impairment in ART-treated PLWH.

## 5. Manifestations of CNS Infection and Damage

### 5.1. BBB Disruption

Disruption of the BBB is a hallmark indicator of CNS injury in HIV infection and reflects a profound breakdown in neurovascular homeostasis. HIV induces BBB deterioration through a combination of direct viral protein toxicity, endothelial dysfunction, and immune-mediated inflammatory signaling, each of which contributes to increased permeability and sustained neuroinflammation.

### 5.2. HIV Protein Effects

Viral proteins, primarily gp120 and Tat, directly impair BBB integrity by upregulating matrix metalloproteinases (MMP-2, MMP-9) and promoting the degradation of critical tight-junction proteins such as claudin-5, occludin, and ZO-1 [24,148,166,167,168,169,170]. This enzymatic breakdown weakens the endothelial barrier, enabling infiltration of circulating immune cells and neurotoxic plasma components. Additionally, the accessory protein Vpr exacerbates endothelial damage by inducing oxidative stress, mitochondrial dysfunction, and apoptotic signaling, further destabilizing the microvascular architecture [137,150].

### 5.3. Immune Cell Infiltration

The BBB disruption facilitates the entry of monocytes and macrophages, driven in part by elevated chemokines such as CCL2, CXCL10, and others released by activated microglia and astrocytes [137,148]. Once within the CNS, these infiltrating cells expand the reservoir of HIV-target cells and amplify local cytokine production. This establishes a positive feedback loop wherein immune cell infiltration enhances inflammation, and inflammation further deteriorates the BBB. In parallel, peripheral CD8^+^ T cells can also penetrate the compromised BBB. Although critical for antiviral defense, these cells release granzyme B and perforin, which inflict bystander neuronal injury and contribute to synaptic degeneration [137,171].

Thus, once the barrier becomes increasingly permeable, the CNS is exposed to a range of circulating neurotoxins, including viral proteins, inflammatory cytokines, and plasma-derived components such as fibrinogen, which are ordinarily excluded from the brain parenchyma damage [18,148]. These molecules further activate glial cells, intensify oxidative stress, and perpetuate neuronal injury, creating a self-reinforcing cycle of neurovascular dysfunction and neuroinflammation.

### 5.4. Chronic Immune Activation

Persistent immune activation in the CNS, driven by HIV proteins, activated glia, and infiltrating immune cells, creates a neurotoxic environment that progressively undermines neuronal integrity and cognitive function. This chronic inflammatory milieu promotes bystander neuronal injury, disrupts neurotrophic support, and accelerates maladaptive synaptic remodeling, collectively contributing to the development and persistence of HAND.

### 5.5. Bystander Neuronal Injury

Pro-inflammatory cytokines such as TNF-α and IL-1β, released by activated microglia and astrocytes, directly impair synaptic plasticity, alter neurotransmission, and promote dendritic spine retraction and pruning [150,172]. These cytokines sensitize neurons to excitotoxic damage by modulating glutamatergic signaling and weakening antioxidant defenses. Concurrently, glia-derived ROS and excessive extracellular glutamate induce lipid peroxidation, mitochondrial depolarization, and DNA damage, culminating in intrinsic apoptotic signaling [24,150]. This constellation of inflammatory and oxidative insults drives progressive neuronal atrophy and functional decline.

### 5.6. Loss of Neurotrophic Support

Neuronal survival and synaptic integrity depend on adequate levels of BDNF, which become markedly reduced in the context of chronic HIV infection and neuroinflammation. Diminished BDNF correlates strongly with synaptic loss, disrupted plasticity, and cognitive impairment observed in PLWH [137,172]. Compounding this deficit, the accumulation of proBDNF, the precursor form of BDNF, activates p75NTR receptors, triggering caspase-3-dependent apoptotic pathways and further exacerbating neuronal loss [172]. Thus, impaired neurotrophic signaling constitutes a critical mechanism linking inflammation to neurodegeneration.

### 5.7. Excessive Synaptic Pruning

Microglia contribute to neurodegeneration through overactive synaptic pruning, a process normally involved in developmental refinement. Activated microglia upregulate complement and chemokine pathways, including the CX3CL1-CX3CR1 axis, which facilitates targeting and engulfment of synaptic elements [137,172]. In the inflamed HIV-infected brain, this pruning becomes maladaptive, leading to loss of dendritic spines, weakening of neural networks, and impaired cognitive processing. Over time, excessive microglial phagocytosis of synapses contributes significantly to structural and functional connectivity deficits characteristic of HAND.

### 5.8. Perpetual Viral Protein Production and Dissemination

Despite the profound success of ART in suppressing plasma viremia, ART regimens do not directly inhibit HIV transcription. As a result, low-level proviral transcription persists in PLWH, even during long-term viral suppression. This transcription originates not only from intact, replication-competent proviruses, but also from defective proviruses that retain a functional LTR promoter, enabling the expression of viral RNA and proteins without producing infectious virions [2,7,46,173]. These persistent transcriptional activities contribute to ongoing immune activation and neurotoxicity within the CNS.

Even under stringent viral suppression, neurotoxic proteins, particularly Tat and gp120, remain detectable in the CSF and brain tissue, indicating continuous release from CNS-resident cells [18,174]. Tat and gp120 exert potent neurotoxic effects by activating glial cells, disrupting synaptic signaling, impairing calcium regulation, and promoting glutamate-mediated excitotoxicity. Importantly, HIV proteins can also be transported via EVs, which carry functional Tat, Nef, and other viral factors into neurons and glial cells [149,150]. These EV-mediated transfers impair autophagy, mitochondrial dynamics, and lysosomal function, thereby amplifying neuroinflammation and accelerating neuronal injury.

### 5.9. Compartmentalized HIV Evolution

The CNS represents a unique anatomical and immunological niche in which HIV can undergo compartmentalized evolution, diverging genetically from circulating peripheral strains. CNS-derived variants often display macrophage-tropism, optimized for infection of microglia and perivascular macrophages, and exhibit enhanced resistance to innate immune responses or inflammatory stressors neurovirulence [18,148]. These adaptations can increase neurovirulence and contribute to persistent neuroinflammation, even when systemic viral replication is fully suppressed [26,175,176]. The persistent low-level HIV transcription, ongoing viral protein dissemination, and compartmentalized viral evolution within the CNS collectively create a chronic pathogenic backdrop that sustains neuroinflammation, disrupts neuronal homeostasis, and contributes to the long-term neurocognitive complications observed in PLWH.

## 6. ART-Associated Neurotoxicity

Although ART has revolutionized HIV care, several antiretroviral drugs, particularly nucleoside reverse transcriptase inhibitors (NRTIs), protease inhibitors (PIs), and some non-nucleoside reverse transcriptase inhibitors (NNRTIs), have been implicated in neurotoxicity and mitochondrial dysfunction [76,177,178,179,180,181,182]. Chronic exposure to these drugs leads to oxidative stress, altered calcium signaling, and neuronal apoptosis. For instance, efavirenz, an NNRTI widely used in first-line therapy, induces mitochondrial depolarization, impairs respiratory chain function, and promotes activation of CNS cells, mainly microglia and astrocytes, due to the generation of ROS and other reactive free radical species and NF-κB activation [178,180,183,184,185]. Similarly, PIs such as lopinavir/ritonavir interfere with lipid metabolism, disrupt endoplasmic reticulum function, and impair autophagy, all contributing to neuronal injury [183,186]. NRTIs are also shown to cause mtDNA depletion and neuronal energy failure, particularly in CNS regions with high metabolic demands.

Notably, ART-induced neurotoxicity is multifactorial and reflects (i) direct drug effects on CNS-resident cells, (ii) indirect neuroinflammatory/vascular effects, and (iii) pharmacokinetic factors (CNS penetration, intracellular accumulation, transporter interactions). Importantly, neurotoxicity risk varies widely by drug class and individual agent, and is influenced by age, comorbidities, and concomitant medications. For example, …

Non-nucleoside reverse transcriptase inhibitors (NNRTIs)—Efavirenz (EFV) is the most consistently linked to neuropsychiatric and neurotoxic phenotypes (vivid dreams, insomnia, mood symptoms; and in experimental systems, neuronal/glial stress). However, older NNRTIs (e.g., nevirapine) have less consistent neurotoxicity signals than EFV [178,180,181,183,184,185,187].

Nucleos(t)ide reverse transcriptase inhibitors (NRTIs)—Specifically, stavudine (d4T), didanosine (ddI), zidovudine (AZT) (and to a lesser extent some others). These are classically associated with mitochondrial toxicity systemically and can contribute to CNS bioenergetic stress. Interestingly, the newer NRTIs generally show improved safety profiles, but mitochondrial effects can still be detected in some experimental paradigms [181].

Protease inhibitors (PIs)—Some PIs (e.g., lopinavir/ritonavir, and historically, indinavir) are associated with metabolic and inflammatory disturbances that can secondarily impact the brain; direct CNS cell stress has also been reported in preclinical models for select agents [180,181,182].

Integrase strand transfer inhibitors (INSTIs)—INSTIs are widely used and generally well tolerated, but neuropsychiatric symptoms (sleep disturbance, anxiety/depression in a subset) have been reported for some agents, most often discussed for dolutegravir and sometimes bictegravir, with mechanisms still being clarified and likely heterogeneous [181,182,187].

Entry/attachment/fusion inhibitors and CCR5 antagonists—Less frequently implicated in direct neurotoxicity overall; CNS effects are typically discussed more in the context of efficacy/penetration than intrinsic toxicity (agent-dependent) [181,182,188].

### Mechanisms Implicated for Persistent CNS Inflammation Under Suppressive ART

Despite systemic viral suppression, multiple studies report persistent low levels of HIV and inflammation in the cerebrospinal fluid (CSF) and brain tissue of ART-treated individuals [183,189,190]. Elevated CSF levels of neopterin, CXCL10, and sCD163 persist even in aviremic patients, indicating ongoing microglial activation and macrophage turnover [190,191]. These residual HIV and inflammatory signals may arise from incomplete ART penetration into the CNS, long-lived infected macrophages, and continued release of viral proteins and EVs.

Another emerging contributor is ART-induced mitochondrial and lysosomal dysfunction, which can activate inflammasomes such as NLRP3 and AIM2, thereby sustaining glial activation [192,193]. Chronic exposure to ART can also promote senescence-like phenotypes in astrocytes and microglia, marked by upregulation of p21, Senescence-Associated Secretory Phenotype (SASP) cytokines, e.g., IL-6, and NF-κB signaling, which mimic HIV-induced inflammatory effects [194].

Furthermore, ART itself alters CNS lipid metabolism and mitochondrial homeostasis, leading to increased ceramide accumulation and oxidative stress, pathways that synergize with viral protein toxicity. These overlapping mechanisms underscore the concept of a “two-hit model” of NeuroHIV pathology: (1) primary insult from HIV infection and viral proteins, and (2) secondary injury from long-term ART exposure and aging-related oxidative stress [177].

In summary, neuroinflammation in HIV represents a multifaceted process sustained by viral persistence and its gene products, aging-associated inflammation, epigenetic deregulation, and ART-induced cellular stress. Understanding these molecular cross-talks offers promising therapeutic entry points for interventions aimed at mitigating both neuroinflammation and accelerating the acquisition of age-associated comorbidities.

## 7. Epigenetic Regulation in HIV-Associated Neuroinflammation

Epigenetic dysregulation has emerged as a mechanism linking chronic HIV persistence, neuroinflammation, and neuronal injury in the CNS. HIV infection alters the chromatin landscape of microglia and astrocytes by modulating epigenetic changes at gene promoters and enhancers; the changes include DNA methylation, histone modifications, and nucleosome reorganization, leading to sustained transcriptional activation of inflammatory pathways and impaired homeostatic gene expression.

HIV-associated epigenetic dysregulation usually converts transcriptionally suppressive heterochromatin structures to transcription-supporting euchromatin structures at gene promoters, facilitating the sustained production of viral transcripts and inflammatory mediators, as well as modulating the expression of human endogenous retroviruses (HERVs).

Chronic inflammatory signaling and oxidative stress also promote epigenetic drift, a phenomenon characterized by global alterations in DNA methylation and chromatin accessibility, which are typically associated with aging but may occur prematurely in PLWH. These changes disrupt the normal transcription of genes involved in synaptic maintenance, neuroprotection, and metabolic stability. In parallel, loss or post-translational inactivation of TRIM28 through DNA-PK stimulation leads to depression of HERVs, whose reactivation can stimulate TLR and cGAS-STING pathways, further amplifying neuroinflammation [3,195,196,197,198,199].

Thus, epigenetic dysregulation acts as both a driver and a consequence of chronic HIV-induced neuroinflammation, creating a self-sustaining loop that maintains viral persistence and glial activation within the CNS microenvironment.

## 8. HIV, Neurotoxicity, Aging, and Multimorbidity

The demographic landscape of HIV infection has undergone a profound transformation over the past two decades. Advances in ART have dramatically improved survival rates, resulting in an aging population of PLWH. Especially in many high-income countries, over half of the HIV-positive population is now aged 50 years or older. This shift reflects both the improved longevity resulting from effective treatment and the ongoing emergence of new infections among older adults. As a result, clinicians and researchers face another challenge pertaining to managing the complex health needs of aging PLWH [13,22,200,201,202].

Aging in the context of HIV is primarily characterized by accelerated or accentuated neurodegeneration. In addition, aging with HIV is associated with a significantly higher burden of comorbidities compared to HIV-negative peers. These include cardiovascular disease, metabolic disorders such as diabetes, chronic kidney disease, osteoporosis, and non-AIDS-defining cancers. The prevalence of these conditions is often higher and occurs at an earlier age among PLWH than in the general population [18]. Polypharmacy is common due to the need to manage both HIV and multiple comorbid conditions, which increases the risk of drug–drug interactions and adverse effects. Additionally, frailty and geriatric syndromes, such as cognitive impairment, falls, and functional decline, occur at higher rates and present at younger ages in PLWH. The complex interplay of HIV infection, chronic immune activation, and aging processes contributes to this increased vulnerability, necessitating comprehensive, multidisciplinary care approaches.

HIV infection accelerates several key aspects of biological aging. Immunosenescence, characterized by a decline in immune function and altered T cell phenotypes, is observed prematurely in PLWH [22]. This is evidenced by increased proportions of senescent CD8+ T cells and a reduced pool of naïve T cells. Epigenetic aging, as measured by DNA methylation clocks, demonstrates accelerated aging signatures in HIV-infected individuals, with brain tissue in particular showing epigenetic age acceleration of several years compared to uninfected controls.

Chronic immune activation, driven by microbial translocation, residual HIV transcription, and persistent glial reactivity, produces an “inflammaging” phenotype marked by systemic elevated IL-6, TNF-α, CRP, IL-17, and sCD14 levels. This inflammatory burden accelerates vascular pathology, promotes BBB permeability, and heightens oxidative stress, all of which contribute to the early onset of cerebrovascular small vessel disease (CSVD) and increased cognitive decline [142,161]. Mitochondrial dysfunction is also prominent, driven by both HIV proteins and certain antiretroviral drugs, leading to impaired mitochondrial DNA replication, increased oxidative stress, and energy deficits. Moreover, age-associated reductions in BDNF and other neurotrophic factors weaken neuronal survival pathways, making neurons more susceptible to inflammatory and oxidative insults. Together, these conditions promote synaptic degeneration, microglial dysregulation, and white matter abnormalities [22,203,204,205]. These aging-associated deficiencies contribute to the early onset of age-related diseases and neurocognitive decline in PLWH [13,22,161,200,201,202,206,207,208].

The intersection of HIV infection, chronic inflammation, and aging creates a synergistic environment that amplifies the risk of multiple age-related conditions [209]. Persistent immune activation and systemic inflammation, even under suppressive ART, drive endothelial dysfunction, atherosclerosis, and an increased risk of cardiovascular disease [13,22,161,208]. Neuroinflammation contributes to the high prevalence of HAND, with aging further exacerbating synaptic loss and brain atrophy. Chronic inflammation also promotes insulin resistance, bone demineralization, and frailty [210,211,212,213]. This convergence of factors complicates clinical management, requiring integrated approaches to address multimorbidity, polypharmacy, and functional decline in aging PLWH [214,215].

In summary, HIV and aging converge on overlapping pathways, including chronic inflammation, mitochondrial dysfunction, vascular injury, and epigenetic drift, to amplify neurodegenerative processes beyond what would be expected from chronological aging alone.

## 9. Prominent Indicators of Accelerated Aging in PLWH

### 9.1. Chronic Inflammation (“Inflammaging”)

Neuroimaging studies indicate that people living with HIV exhibit accelerated brain aging, estimated at approximately 3.5 to 7.4 years relative to uninfected controls, which correlates with impairments in motor and executive function. Chronic inflammation represents a shared and defining hallmark of both aging and HIV infection, linking these neurocognitive and structural changes. In aging, systemic and perpetual inflammation termed “inflammaging” involves elevated pro-inflammatory cytokines (e.g., TNF-α, IL-6, IL-18, IL-1β) and immune senescence. HIV amplifies this process through:

### 9.2. Persistent Immune Activation

Despite effective ART, PLWH often exhibit chronic systemic and CNS immune activation. This persistent inflammatory state is fueled by several interrelated mechanisms, including ongoing low-level HIV gene expression, residual viral replication in anatomical sanctuaries such as the gut and CNS, and microbial translocation due to compromised intestinal mucosal integrity [216,217,218]. Even in the absence of detectable viremia, HIV-derived proteins such as Tat and Nef can be actively expressed from latent or defective proviruses, sustaining immune activation. Additionally, microbial products such as lipopolysaccharide (LPS) from translocated gut bacteria enter the circulation and engage pattern recognition receptors on innate immune cells, further amplifying pro-inflammatory signaling [2,18,21,219,220]. This sustained activation not only contributes to systemic inflammation but also drives neuroinflammation and neurodegeneration through cytokine release and immune cell recruitment into the CNS.

### 9.3. Microglial Dysregulation

Microglia, the brain’s resident innate immune cells, are both targets and mediators of HIV neuropathogenesis. In the setting of HIV infection, microglia become chronically activated and exhibit a dysregulated, pro-inflammatory phenotype [221,222,223]. Infected or activated microglia secrete a range of neurotoxic mediators, including TNF-α, IL-1β, and ROS, all of which contribute to oxidative stress, synaptic pruning, and neuronal injury. This pro-inflammatory activation persists despite systemic viral suppression and is further exacerbated by aging and comorbid factors such as substance use [45,224]. Dysregulated microglial activity not only perpetuates a cycle of inflammation and neuronal damage but also interferes with normal synaptic maintenance and plasticity, undermining cognitive resilience in PLWH.

### 9.4. Blood–Brain Barrier (BBB) Breakdown

The integrity of the BBB is critical for maintaining CNS homeostasis, yet it is compromised during HIV infection. Viral proteins such as gp120 and Tat have been shown to disrupt BBB function by upregulating matrix metalloproteinases (MMPs), particularly MMP-2 and MMP-9, which degrade tight junction proteins (e.g., claudin-5, Occludin, ZO-1) that are essential for barrier maintenance [32,128,225,226]. This enzymatic degradation increases BBB permeability, facilitating the infiltration of peripheral immune cells, neurotoxins, and viral particles into brain parenchyma. The influx of these peripheral elements accelerates neuroinflammation and amplifies glial activation, creating a feed-forward loop that sustains CNS pathology. Notably, ART drugs may not fully reverse BBB disruption, and in some cases, may contribute to vascular dysfunction, highlighting the need for adjunctive therapies targeting BBB repair and neurovascular health [2,18].

### 9.5. Defective Proteostasis

Accelerated biological aging in people living with HIV (PLWH) is increasingly linked to defective proteostasis, a breakdown in the cellular systems that regulate protein synthesis, folding, and degradation. Both aging and chronic HIV infection disrupt proteostasis machinery, leading to the accumulation of misfolded and aggregation-prone proteins that contribute to neurodegeneration [203,217,227,228]. A prominent example is amyloid-β (Aβ) accumulation, which is accelerated in HIV. The viral protein Tat inhibits neprilysin, a major Aβ-degrading enzyme, and can directly cross-seed Aβ fibrils, promoting plaque formation. Postmortem analyses show striking co-localization of HIV proteins with Aβ plaques in cortical tissue of PLWH, reinforcing the overlap between HAND and Alzheimer’s disease-like pathology. Additionally, HIV infection promotes tau hyperphosphorylation through Tat-mediated activation of kinases such as GSK-3β and CDK5, catalyzing the formation of neurofibrillary tangles. Another aging-related proteinopathy, α-synuclein aggregation, is also enhanced in HIV, with studies showing that HIV-1 accelerates α-synuclein fibril formation; these aggregates further facilitate viral entry into microglia and T cells, creating a pathological feedback loop between infection and neurodegeneration. Compounding these effects, HIV proteins, including Nef and Tat, impair autophagy-lysosomal pathways by blocking autophagosome-lysosome fusion, resulting in the buildup of damaged mitochondria and toxic protein aggregates.

Together, these proteostatic disturbances represent key biomarkers of accelerated aging in PLWH and highlight molecular intersections between HIV neuropathogenesis and other classic neurodegenerative diseases.

### 9.6. Mitochondrial Dysfunction

Mitochondria are a major source of ROS and key regulators of innate immune signaling, and their dysfunction amplifies inflammation within the central nervous system. Damaged mitochondria are known to mediate the production of pro-inflammatory cytokines and chemokines [101,179,229,230,231,232,233,234,235,236,237]. Notably, mitochondrial dysfunction is a defining pathological feature of HAND, and is a critical mediator linking viral persistence, chronic inflammation, epigenetic aging, and neurodegeneration in PLWH [80,185,238]. In the CNS, mitochondrial injury arises from convergent upstream insults, including sustained exposure to HIV proteins, chronic immune activation, and chronic ART toxicity; most of them converge on lysosomal dysfunction, which precedes and amplifies mitochondrial damage [22,103,126,181,206,207,228,238,239,240,241,242,243,244,245,246,247,248]. These processes act across multiple CNS cell types, including neurons, microglia, astrocytes, and brain endothelial cells, resulting in impaired bioenergetics, excessive reactive species generation, inflammasome activation, and ultimately neuronal loss [22,205,207,228,239,240,241,242,243]. The mitochondrial dysfunction in different cell types results in neuronal energy deficits, synaptic loss, and oxidative damage to lipids/DNA. Especially, PLWH exhibit shortened telomeres and generation of senescent cells, marking mitochondrial compromise, for example…

HIV protein-mediated mitochondrial toxicity: Certain HIV proteins exert significant mitochondrial toxicity by directly disrupting mitochondrial structure and function, which contributes to neuronal dysfunction and CNS deterioration. The envelope protein gp120 and the transactivator protein Tat disrupt the integrity and function of the electron transport chain (ETC) by impairing key respiratory complexes, leading to increased production of ROS and a concomitant reduction in ATP generation and calcium dysregulation, thereby promoting oxidative stress and energetic failure in neurons and glial cells [79,81,91,101,245]. Another viral protein, Vpr, exacerbates mitochondrial injury by binding directly to mitochondrial membranes and inducing the opening of the mitochondrial permeability transition pore (mPTP), resulting in the release of cytochrome c and activation of intrinsic apoptotic pathways [81,99,101,245,249]. Importantly, these effects occur even in the absence of productive viral replication, underscoring how persistent low-level HIV transcription and extracellular dissemination of viral proteins can sustain mitochondrial injury despite virologic suppression on ART.

ART-induced mitochondrial toxicity: ART further compounds mitochondrial dysfunction. Briefly, certain ART drugs, particularly nucleoside reverse transcriptase inhibitors (NRTIs), contribute additional mitochondrial toxicity by inhibiting mitochondrial DNA polymerase γ, leading to mtDNA depletion, disrupted oxidative phosphorylation, and further impairment of cellular energy metabolism [80,181,238]. Together, HIV protein-mediated mitochondrial disruption and ART-associated toxicity synergistically compromise neuronal survival and exacerbate CNS inflammation and neurodegeneration in people living with HIV [81,99,100,101,245]. Notably, these ART-related effects are not neuron-restricted; other CNS cells, such as astrocytes and microglia, exhibit altered mitochondrial morphology, reduced spare respiratory capacity, and heightened ROS generation following ART exposure, which in turn amplifies neuroinflammatory signaling and excitotoxic injury to neurons.

Lysosomal Dysfunction: Notably, mounting evidence indicates that lysosomal dysfunction lies upstream of mitochondrial injury in HIV-associated neurotoxicity and ART-related adverse effects, acting as a critical but underappreciated initiator of bioenergetic failure. HIV proteins such as Tat, Nef, and gp120 directly impair lysosomal acidification, autophagosome-lysosome fusion, and proteolytic capacity, leading to defective clearance of damaged mitochondria through mitophagy [81,92,101,103,126,245,250]. Similarly, several antiretroviral agents, particularly older NRTIs and some protease inhibitors, disrupt lysosomal membrane integrity, alter intralysosomal pH, and inhibit cathepsin activity, resulting in accumulation of dysfunctional organelles and undegraded protein aggregates [99,100,251,252,253,254,255,256,257,258,259]. Failure of lysosomal quality control permits persistence of damaged mitochondria that generate excessive reactive species, amplifying oxidative stress, mitochondrial DNA damage, and electron transport chain (ETC) dysfunction. It was noted that mitochondrial dysfunction is downstream of lysosomal failure and inflammasome signaling, rather than an isolated defect. Lysosomal dysfunction during HIV infection plays a crucial role in promoting inflammation by activating inflammasomes such as NLRP3 and AIM2 [260,261]. This lysosome-mitochondria-inflammasome axis establishes a vicious cycle: inflammasome activation worsens mitochondrial dysfunction through cytokine-mediated nitric oxide and ROS production, while damaged mitochondria further amplify inflammasome signaling. Persistent NLRP3 activation has been linked to synaptic loss, neuronal death, and cognitive impairment in HAND [102,262]. Briefly, in neurons, combined lysosomal–mitochondrial dysfunction drives ATP depletion, impaired axonal transport, synaptic degeneration, and apoptotic signaling. In microglia, defective mitophagy and mitochondrial ROS sustain a pro-inflammatory, neurotoxic phenotype characterized by persistent cytokine release and inflammasome activation. Astrocytes exhibit impaired glutamate uptake and redox buffering due to mitochondrial dysfunction, exacerbating excitotoxic injury. Brain endothelial cells show mitochondrial stress that compromises tight junction integrity, reinforcing BBB breakdown and immune cell infiltration. Importantly, lysosomal membrane permeabilization also releases redox-active iron and cathepsins into the cytosol, further exacerbating mitochondrial depolarization and triggering apoptotic cascades [90,98].

Additionally, Lysosomal dysfunction is also mechanistically linked to inflammasome activation, providing a direct bridge between organellar stress and chronic neuroinflammation. Lysosomal membrane permeabilization and leakage of cathepsins (e.g., cathepsin B), together with mitochondrial ROS and ionic flux (K^+^ efflux), are recognized triggers that promote NLRP3 inflammasome assembly. In HIV-associated CNS inflammation, microglial NLRP3 activation and IL-1β/IL-18 signaling have been implicated as key amplifiers of synaptotoxic inflammation, and HIV protein exposure (including Tat) can engage these pathways [149,192]. Thus, lysosomal injury is not only upstream of mitochondrial dysfunction via impaired mitophagy, but also upstream of NLRP3-driven neuroinflammatory amplification. This suggests that restoring lysosomal throughput can leave the dominant bottleneck unchanged, that is, damaged mitochondria continue to accumulate because they cannot be removed. This concept is consistent with broader neurodegeneration biology, where lysosomal insufficiency drives secondary mitochondrial failure and synaptic vulnerability.

Given that lysosomal decline is a hallmark of mitochondrial failure, synaptic vulnerability, and brain aging, boosting lysosomal competence is a plausible approach, as it targets HIV/ART proteotoxicity and addresses age-associated loss of proteostasis, a vital link to accelerated/accentuated aging phenotypes in PLWH. These observations suggest that therapeutic strategies focused solely on mitochondrial protection, such as antioxidants or ETC stabilizers, are unlikely to achieve durable neuroprotection unless upstream lysosomal-autophagy pathways are concurrently restored. This concept aligns with broader neurodegeneration biology, where lysosomal insufficiency drives secondary mitochondrial dysfunction and synaptic vulnerability. Hence, collective targeting of lysosomal function, autophagic flux, and mitophagy, therefore, represents a necessary and complementary approach to mitigating HIV- and ART-induced mitochondrial dysfunction and neuronal injury. Such approaches are particularly relevant in aging PLWH, where baseline declines in lysosomal and mitochondrial resilience synergize with HIV- and ART-mediated stressors.

### 9.7. DNA Damage and Epigenetic Alterations

HIV accelerates molecular aging through genomic and epigenetic mechanisms [160,182,185,186], for example, …

Oxidative DNA damage: Chronic HIV infection, persistent neuroinflammation, and mitochondrial dysfunction collectively contribute to substantial oxidative DNA damage within the CNS. Elevated levels of mitochondrial-derived ROS induce single- and double-strand DNA breaks that overwhelm intrinsic repair pathways, including the base excision repair (BER) machinery that normally resolves oxidative lesions. When these repair systems become saturated or impaired, DNA damage accumulates, leading to genomic instability, activation of stress-response pathways, and progressive cellular dysfunction.

Epigenetic aging: In parallel, HIV accelerates epigenetic aging, as evidenced by alterations in DNA methylation patterns measured by established epigenetic clocks. Studies have shown that HIV infection can advance the epigenetic age of brain tissue by 3.5 to 7.4 years, reflecting premature cellular aging. These methylation changes disproportionately affect genes involved in dopamine metabolism, such as COMT and critical components of oxidative phosphorylation, further linking epigenetic dysregulation to mitochondrial deficits and neuronal vulnerability. Together, oxidative DNA damages and accelerated epigenetic aging form a synergistic mechanism that exacerbates neurodegeneration and cognitive decline in people living with HIV.

### 9.8. Structural Changes

As detailed above, functionally compromised lysosomal compartments, impaired autophagic flux, and accumulation of damaged mitochondria with fragmented networks and reduced cristae integrity. Neuronal susceptibility is heightened because synapses and long axons depend on efficient lysosomal trafficking and local mitophagy to maintain bioenergetic stability. In HIV-relevant contexts, Tat-associated neuronal injury involves lysosomal dysfunction (supporting lysosomes as an early structural/functional lesion) [81,101,103,245]. The downstream, persistent mitochondrial dysfunction, driven by reactive species, calcium stress, and inadequate clearance, creates the morphological substrate for reduced ATP buffering at synapses, and progressive loss of connectivity leads to primarily accelerated white matter loss in the caudate, hippocampus, and prefrontal cortex in older PLWH compared to age-matched HIV-negative controls [263,264].

White matter loss: White matter integrity is profoundly affected in PLWH, even in the era of effective ART [265,266]. Neuroimaging studies consistently reveal diffuse white matter hyperintensities (WMH) and reductions in fractional anisotropy (FA) within frontal, subcortical tracts, regions highly vulnerable to neuroinflammation and neurovascular injury. These abnormalities are strongly associated with microglial activation, chronic immune-mediated myelin damage, and BBB disruption, which together impair axonal conduction and degrade structural connectivity across neural networks. Importantly, aging interacts synergistically with HIV to exacerbate white matter decline. Individuals with HIV aged 50 years or older exhibit WMH volumes and microstructural changes comparable to HIV-negative adults nearly a decade older, highlighting an accelerated or accentuated aging phenotype in HIV [267]. This premature white matter deterioration contributes significantly to cognitive slowing, executive dysfunction, and the broader neurocognitive impairments characteristic of HAND.

Synaptic degeneration: Synaptic degeneration is a hallmark of HIV-associated neurocognitive decline and reflects the cumulative impact of chronic inflammation and viral protein toxicity on neuronal circuits. Postmortem analyses of brains from people living with HIV reveal pronounced dendritic pruning, loss of dendritic spines, and significantly reduced synaptic density within the frontal cortex, an area crucial for executive function, attention, and working memory. These structural abnormalities arise from the persistent activation of microglia and astrocytes, which release pro-inflammatory cytokines and complement factors that tag synapses for elimination. Concurrently, HIV proteins such as Tat and gp120 directly disrupt synaptic signaling, impair glutamatergic balance, and induce calcium dysregulation, further destabilizing synaptic connections. Together, these inflammatory and viral mechanisms converge to weaken neuronal communication, degrade network connectivity, and drive the cognitive and behavioral impairments characteristic of HAND.

Cerebrovascular disease: Cerebrovascular dysfunction is increasingly recognized as a significant contributor to neurological morbidity in people living with HIV, even in the context of effective viral suppression. Numerous imaging and pathological studies reveal a heightened prevalence of silent cerebral small vessel disease (CSVD), including microinfarcts, microbleeds, and perivascular space enlargement, which often occur independently of traditional vascular risk factors such as hypertension or diabetes. HIV-related chronic inflammation, endothelial activation, and persistent immune dysregulation contribute to non-atherosclerotic vasculopathies characterized by arterial wall thickening, impaired vasoreactivity, and abnormal vascular remodeling. These changes compromise cerebral blood flow and impair neurovascular coupling, further exacerbating neuronal vulnerability and accelerating cognitive decline. The increased burden of CSVD in HIV not only reflects direct viral and inflammatory effects on the cerebral microvasculature but also highlights a broader susceptibility to vascular injury, reinforcing the intersection between HIV infection, aging, and neurodegenerative processes [161,205,268,269,270,271].

Overall, a growing body of evidence indicates that HIV infection and biological aging converge on several interrelated pathogenic pathways, namely, chronic inflammation, impaired proteostasis, mitochondrial dysfunction, and epigenetic dysregulation, which collectively accelerate neurodegenerative processes and contribute to premature or accentuated brain aging in PLWH. These overlapping mechanisms create a neurobiological environment that favors synaptic loss, impaired neurogenesis, white matter abnormalities, and cognitive decline. While ART has been highly effective in suppressing peripheral viremia and extending lifespan, it does not fully prevent or reverse neurocognitive deterioration. Persistent low-level viral activity in the CNS, alongside ART-induced neurotoxicity and incomplete immune reconstitution, further exacerbates brain injury. As a result, many aging PLWH continue to experience HAND, which profoundly affects quality of life, functional independence, and healthcare burden.

To address this unmet clinical need, there is an urgent call for adjunctive therapies that specifically target the cellular and molecular drivers of HIV- and age-related neurodegeneration. These may include agents aimed at dampening neuroinflammation, restoring mitochondrial function, enhancing protein clearance mechanisms (e.g., autophagy), or reversing epigenetic dysregulation. Thus, aging PLWH faces a “double hit” of HIV-driven and age-related neurodegeneration, marked by protein aggregation, mitochondrial failure, and structural brain changes [205,271]. These processes culminate in higher rates and severity of HAND, with overlapping features of Alzheimer’s and Parkinson’s pathologies. Addressing these mechanisms requires therapies targeting both viral persistence and aging; future therapeutic strategies must incorporate CNS-penetrant compounds capable of reaching viral reservoirs and modulating the neuropathogenic environment directly. Prioritizing the development and deployment of such neuroprotective interventions, guided by precision medicine approaches and supported by innovative preclinical models, will be essential to mitigate HAND and improve neurocognitive outcomes for the growing population of older adults living with HIV.

## 10. Future Directions

### 10.1. Novel Tools and Prominent Molecular Pathways

As a substantial proportion of PLWH, particularly in high-income countries, now survive into older age, a major priority for the field is to identify and therapeutically target the mechanisms driving accelerated or accentuated aging in this population. Advancing this goal requires moving beyond descriptive associations to uncover causal biological pathways connecting chronic HIV infection, low-level viral persistence, sustained neuroinflammation, cerebrovascular injury, epigenetic dysregulation, and multisystem comorbidities. Achieving this will necessitate longitudinal, deeply phenotyped cohorts that integrate clinical assessments with neurocognitive testing, advanced neuroimaging, immunologic and virologic profiling, and comprehensive multi-omics datasets, including epigenomic, transcriptomic, proteomic, and metabolomic measures. Such integrative approaches will be essential for defining heterogeneous aging trajectories among PLWH, identifying modifiable drivers of biological aging, and informing precision therapeutic strategies aimed at preserving cognitive and functional health across the lifespan.

At the mechanistic level, there is an urgent need to clarify how persistent HIV transcription and viral protein expression intersect with host aging pathways, including mitochondrial dysfunction, oxidative stress, cellular senescence, and epigenetic drift. Particular emphasis should be placed on dysregulated DNA damage response (DDR) pathways, and how these abnormalities converge with inflammatory and neurodegenerative cascades across microglia, astrocytes, endothelial cells, and neurons. Advanced in vitro platforms, including iPSC-derived microglia, astrocytes, neurons, and tri-lineage cerebral organoids, as well as “aged” or stress-induced aging models, will be critical for dissecting cell-type-specific vulnerabilities and identifying molecular points of intervention. Integrating these systems with single-cell and spatial multi-omics technologies will further enable high-resolution mapping of transcriptional, epigenetic, and metabolic alterations within discrete CNS cell populations. Complementary studies using humanized mouse models and aging-accelerated animal systems will be essential for validating mechanistic insights and experimentally manipulating these pathways in vivo, ultimately guiding the development of targeted therapies to mitigate HIV-associated accelerated aging.

### 10.2. Therapeutic Implications

Suppressing perpetual HIV transcription: Develop strategies for transcriptional silencing of HIV to reduce viral protein production (Tat, gp120) and diminish chronic CNS inflammation. This would require supplementing ART with transcriptional inhibitors.

Host-directed therapies: Target inflammatory pathways (e.g., NLRP3 and AIM2 inflammasome), cellular senescence (SASP factors), and epigenetic regulators (TRIM28). Develop small molecules or biologics that modulate dysregulated immune activation in the aging HIV-infected brain.

Neurovascular protection: Create therapies that preserve BBB integrity, stabilize endothelial junctional complexes, and reduce CSVD. Enhance cerebrovascular perfusion and reduce ischemic burden in aging PLWH.

Mitochondrial and metabolic resilience: Advance interventions that support mitochondrial biogenesis, reduce oxidative stress, and improve neuronal/glial energy metabolism (e.g., NAD^+^ boosters, antioxidants, OXPHOS stabilizers). Evaluate strategies to reverse HIV- and ART-induced mitochondrial DNA depletion.

Targeting the greater lysosomal system: Ongoing and clinically relevant therapeutic strategies increasingly recognize the “greater lysosomal system” as a central upstream regulator of neurotoxicity and aging in people living with HIV. Large clinical trials such as REPRIEVE demonstrate that reducing chronic inflammation with statins (e.g., pitavastatin) lowers systemic immune activation, indirectly alleviating lysosome-to-mitochondria stress. Inflammasome-adjacent approaches, including IL-1 pathway inhibition (e.g., anakinra) and low-dose colchicine, provide early clinical evidence that dampening innate immune signaling can modify downstream organelle dysfunction in PLWH. Beyond inflammation, autophagy- and lysosome-enhancing strategies, aimed at restoring lysosomal acidification, improving autophagosome-lysosome fusion, stabilizing lysosomal membranes, and promoting mitophagy, are emerging from aging and neurodegeneration research. These approaches are particularly relevant because certain HIV proteins and some ART drugs initiate stress at the lysosomal level, with mitochondrial injury occurring secondarily [101,103,245].

Optimizing ART for the aging brain: Prioritize ART regimens with reduced mitochondrial, metabolic, and neuropsychiatric toxicity. Systematically assess long-acting injectable ART (LA-ART) in older adults. Incorporate CNS outcomes, neurocognitive performance, advanced MRI markers, and CSF/plasma biomarkers into ART selection algorithms.

Advanced modeling and mechanistic studies: Use iPSC-derived microglia, neurons, astrocytes, and multi-lineage cerebral organoids, including “aged” models, to dissect cell-specific vulnerabilities. Integrate single-cell and spatial multi-omics to map aging pathways at high resolution. Validate findings in humanized and aging-accelerated animal models.

Precision aging and clinical integration: Build longitudinal, multi-omics cohorts to identify biological drivers of accelerated aging in PLWH. Develop risk prediction tools and biomarkers for early detection of cognitive decline, vascular injury, and frailty. Promote integrated HIV-geriatrics-neurology care models tailored for older PLWH.

In summary, from a clinical and public health standpoint, future efforts must integrate geriatric, cardiovascular, neurologic, and psychiatric care into routine HIV management to address the complex needs of an aging HIV population. This includes developing and validating screening tools for the early detection of cognitive impairment, frailty, and cerebrovascular disease in PLWH, as well as implementing lifestyle and behavioral interventions that target shared risk factors such as smoking, substance use, physical inactivity, and sleep disturbances. Equally important is the need to confront structural barriers, including stigma, economic disadvantage, and unequal access to healthcare, which exacerbate both HIV- and aging-related vulnerabilities. Particular attention should be directed toward understudied and disproportionately affected groups, including women, racial and ethnic minorities, with specific sexual orientations, individuals with substance use disorders, and people living in low- and middle-income countries, where the intersection of HIV, aging, and resource constraints is especially severe.

Ultimately, the future of HIV care will require a paradigm shift from a narrow focus on viral suppression to a comprehensive strategy that promotes healthy aging with HIV. Achieving this will demand coordinated advances in basic science, translational research, and implementation science to develop interventions that not only control viral replication but also slow biological aging, protect brain and vascular health, and preserve cognitive and functional independence throughout the lifespan of people living with HIV.

## 11. Conclusions

HIV infection remains a uniquely challenging chronic disease due to its capacity to establish long-lived, latent reservoirs in both peripheral tissues and the CNS. These reservoirs are seeded rapidly after infection, are highly resistant to immune clearance, and persist even under long-term, fully suppressive ART. Their inaccessibility, particularly within immune-privileged compartments such as the CNS, continues to impede HIV remission and shapes the long-term clinical trajectory of PLWH.

Neurotoxicity in HIV arises through a combination of direct viral effects and indirect inflammatory mechanisms. Viral proteins, including gp120, Tat, and Nef, disrupt neuronal signaling, impair mitochondrial function, induce excitotoxicity, and trigger apoptotic pathways [81,101,245]. Simultaneously, HIV inflicts indirect neurotoxicity via chronic neuroinflammation driven by glial activation, oxidative stress, and BBB dysfunction, together promoting synaptodendritic injury, network disconnection, HAND, and accelerated/accentuated brain aging. Together, direct and indirect neurotoxicity mechanisms destabilize the CNS milieu due to chronic neuroinflammation involving microglial and astrocytic activation, persistent immune signaling, oxidative stress, and blood–brain barrier dysfunction. Together, these processes contribute to widespread synaptic injury and neuronal loss, underpinning the high burden of HAND and the phenomenon of accelerated or accentuated brain aging in PLWH.

To strengthen mechanistic inference beyond association, future work may also embed causal inference frameworks, including two-sample Mendelian randomization (MR), to test whether modifiable exposures (e.g., inflammatory pathways, metabolic traits, microbiome features, smoking/substance-use proxies) causally influence HIV susceptibility, neuroinflammation, or neurocognitive outcomes, while minimizing confounding and reverse causation. As illustrated in recent HIV-focused MR work, robust inference should incorporate complementary estimators (IVW, weighted median/mode, MR-Egger), tests for heterogeneity and directional pleiotropy (Cochran’s Q, MR-Egger intercept), and sensitivity procedures (leave-one-out, Steiger directionality, and, when appropriate, multivariable MR and phenome-wide pleiotropy screening) to evaluate instrument validity and ensure conclusions are not driven by a subset of variants [272,273].

Although ART has transformed HIV into a manageable chronic illness, it does not fully halt or reverse neurodegeneration. Persistent low-level viral transcription, residual inflammation, incomplete ART penetration into the CNS, and the potential neurotoxicity of certain antiretroviral agents continue to drive CNS injury, particularly in older adults and those with comorbidities such as cardiovascular disease or substance use. These challenges highlight the importance of early diagnosis, timely initiation of ART, careful selection of regimens with minimal neurotoxic potential, and ongoing surveillance for neurocognitive decline.

Looking ahead, progress in HIV management and cure research will require a multifaceted strategy. Novel interventions targeting latent reservoirs, such as latency-reversing agents, gene-editing technologies, and nanoparticle-based delivery platforms, offer promise for reducing or eliminating the persistent viral pool. Complementary neuroprotective approaches, including anti-inflammatory agents, antioxidants, mitochondrial stabilizers, and neurotrophic factor-based therapies, are being explored to mitigate ongoing CNS injury and preserve cognitive function. The integration of personalized medicine, using biomarkers to guide ART selection and identify individuals at the highest risk for neurocognitive decline, will further refine long-term care as the HIV-positive population continues to age.

In summary, the intersection of HIV persistence, neurotoxicity, and aging presents an ongoing clinical and scientific challenge. Yet, rapid advances in reservoir biology, neuroinflammation research, and therapeutic innovation continue to deepen our understanding and expand the possibilities for intervention. Future work must prioritize CNS-penetrant therapies, targeted latency-modulating tools, and durable neuroprotective strategies to improve long-term neurological health and advance the pursuit of a functional cure for HIV.

## Data Availability

The data presented in this study are available in PubMed [https://pubmed.ncbi.nlm.nih.gov].
